# Dormancy cues alter insect temperature–size relationships

**DOI:** 10.1007/s00442-014-3094-4

**Published:** 2014-09-27

**Authors:** Sharon F. Clemmensen, Daniel A. Hahn

**Affiliations:** 1 Department of Ecology and Evolutionary Biology, University of Tennessee, 569 Dabney Hall, Knoxville, TN 37996-1610 USA; 2 Department of Entomology and Nematology, University of Florida, Gainesville, FL USA

**Keywords:** Diapause, Lipid storage, Nutrient allocation, Phenotypic plasticity, Temperature–size rule

## Abstract

Developmental temperatures can have dramatic effects on body size in ectotherms. Thermal plasticity in body size is often viewed in the context of seasonality, but the role of seasonal dormancy responses in generating temperature–size relationships is underappreciated. We used the moth *Helicoverpa zea* (corn earworm) to examine how photoperiodic seasonal dormancy programming for pupal diapause affects the temperature–size relationship. Specifically, we partition out the contributions of somatic growth versus nutrient storage as fat to the thermal reaction norm for size. With increasing temperature from 16 °C to 20 °C, dormant pupae were both overall larger and progressively fatter than non-dormant pupae. This body mass response is likely driven by concurrent increases in food consumption and longer development times as temperatures increase. Our results demonstrate that seasonal photoperiodic cues can alter temperature–size relationships during pre-dormancy development. For biologists interested in seasonal effects on temperature–size relationships, our results suggest that the key to fully understanding these relationships may lie in integrating multiple seasonal cues and multiple aspects of body size and composition in a nutrient-allocation framework.

**Electronic supplementary material:**

The online version of this article (doi:10.1007/s00442-014-3094-4) contains supplementary material, which is available to authorized users.

## Introduction

Body size is a critical aspect of an animal’s life history because it has direct effects on numerous fitness correlates, like survival and fecundity (Nylin and Gotthard 1998; Kingsolver and Huey 2008). Yet plasticity in body size is nearly ubiquitous with body size responding to a myriad of factors, including developmental temperature (Kingsolver and Nagle 2007). The majority of ectotherms, some estimate 80 %, show decreased body sizes as temperature increases, a response termed the “temperature–size rule” (Atkinson 1994). However, some ectotherms show no relationship or even a reversed relationship with some species growing larger at higher temperatures (Atkinson 1994; Blanckenhorn and Demont 2004). The universality of the temperature–size rule, whether observed temperature–size relationships are adaptive, and the proximate physiological mechanisms that produce thermally induced plasticity in size are hotly debated (Van der Have and de Jong 1996; Atkinson and Sibly 1997; de Jong 2010; Watt et al. 2010).

Thermal regimes vary seasonally across generations in multivoltine ectotherms. Some life history models predict that the temperature–size rule is a form of adaptive plasticity that optimizes fitness in this seasonal context (Atkinson 1995; Angilletta and Dunham 2003; Angilletta et al. 2004; Kingsolver et al. 2007; Stillwell et al. 2008; Chown and Gaston 2010). Yet, nearly an equal number of studies suggest that temperature–size relationships are simply a product of non-adaptive developmental processes (Van der Have and de Jong 1996; Angilletta et al. 2004; Davidowitz et al. 2004). Nearly all organisms experience some time during the year when environmental conditions are too stressful for development or reproduction. Many animals have evolved a facultative seasonal dormancy to avoid stressful periods of the year and to time their life cycles to exploit favorable periods (Stearns 1992; Denlinger 2001; Roff 2001). Seasonal dormancy typically includes long periods where feeding is restricted or eliminated. Upon receiving cues that program them for dormancy, animals often alter growth and resource-allocation patterns to increase their nutrient reserves (Hahn and Denlinger 2007, 2011). These greater nutrient reserves have been associated with enhanced survival of dormancy and greater post-dormancy performance (Ishihara and Shimada 1995; Saunders, 2000; Ellers and Van Alphen 2002). Like temperature–size relationships, seasonal plasticity in nutrient reserves is thought to be adaptive (Tauber et al. 1986; Danks 1987). The process of accumulating these additional reserves as part of the dormancy preparatory program may affect body size, growth rates, development time, and feeding (Raubenheimer et al. 2007; Hahn and Denlinger 2007, 2011).

A widely supported model for temperature–size relationships posits that thermal constraints on development time drive organismal temperature–size responses. This model specifically predicts that higher temperatures increase growth rate and decrease development time (Clarke 2003; Walters and Hassall 2006). The interaction of these two factors thereby results in the temperature–size rule if growth rate increases are relatively small compared to decreases in development time (Davidowitz et al. 2004). However, the interaction between temperature, growth rate, and development time can also result in alterations or even a reversal of the temperature–size rule if temperature-dependent increases in growth rate are large relative to decreases in development time (Davidowitz et al. 2004). Differences in photoperiod can also alter the influence of temperature on growth rates (Gotthard et al. 2000). Taken together, these studies suggest that the temperature–size relationship observed may be a product of both adaptive and non-adaptive processes depending on both the taxa used and the environmental context in which the relationship is quantified.

Environmental temperature and seasonality can interact to affect resource acquisition in ectotherms through both effects on diet quality (e.g., host plant nutrients or defenses) and digestive efficiency (Scriber and Slansky 1981; Stamp 1990; Van Asch and Visser 2007; Coggan et al. 2011; Bauerfeind and Fischer 2013; Clissold et al. 2013; Morehouse et al. 2013). Beyond resource acquisition, temperature may also alter allocation to nutrient storage (fat mass) and somatic size (lean mass) independently from each other during development. Thus, it is important to consider the temperature dependence of investment into both the fat mass and lean mass components beyond just the overall body mass relationship when placing temperature–size relationships in a seasonal context (Karl and Fischer 2008). Yet, to our knowledge the importance of seasonal shifts in nutrient allocation between somatic mass and nutrient reserves to temperature–size relationships has not been directly addressed. Here we test whether seasonal cues that trigger a dormancy response, specifically photoperiodic diapause, will alter the temperature–size relationship by altering patterns of nutrient allocation between stored reserves and lean mass. We use an ectotherm with a wide latitudinal distribution and a clear seasonal dormancy response (pupal diapause), *Helicoverpa zea* (Boddie).

## Materials and methods

### Study organism

*Helicoverpa zea*, the corn earworm, is a noctuid moth species in the Heliothinae subfamily. The life cycle of *H. zea* is highly dependent on latitude and the length of the growing season. Individuals generally complete their life cycle in 30 days and can have between one and seven generations per year (Capinera 2001). The high dispersal capabilities of adults lead to a yearly re-colonization of regions that are too cold for *H. zea* to overwinter successfully, higher than 40ºN and 40ºS latitude (Fitt 1989). In areas where *H. zea* successfully overwinters, adults emerge between March and April and begin mating. Larvae are polyphagous and feed most often on plant reproductive structures. Individuals typically have six larval instars (Capinera 2001). After the growing period larvae drop off the host plant, burrow into the soil, and prepare a pupal chamber. In warm temperatures the pupal stage will last 13 days, while individuals in colder temperatures may remain in the pupal stage for over 250 days (Capinera 2001). *H. zea* can use facultative dormancy to avoid poor environmental conditions (Phillips and Newsom 1966), and generally overwinter between 40ºN and 40ºS in North and South America and emerge when temperatures signal the return of conditions suitable for growth (Fitt 1989).

### General rearing methods and parameterizing the photoperiod–diapause response

*H.zea* eggs were obtained from the North Carolina State University Insectary and maintained in a colony at the United States Department of Agriculture (USDA)/Agricultural Research Service-Center for Medical, Agricultural and Veterinary Entomology facility in Gainesville, Florida. The *H. zea* colony at the North Carolina State Insectary was started with moths from North Carolina, a state in which moths experience a yearly overwinter diapause period (Fitt 1989), and periodically renewed with North Carolina wild stock. Experimental eggs were kept at room temperature (21–22 °C) until hatching. After hatching, we reared larvae in groups of 60–70 individuals in 150-mL cups in a 25 °C chamber at 14-h light:10-h day (L:D) (long-day) conditions. Larvae were fed tobacco budworm artificial diet from BioServ (no. F9781B, wheat germ base, Aureomycin antibiotic; Frenchtown, NJ). Upon reaching the third of six instars, clearly discernible by a change in head capsule size and color, larvae were placed individually in 30-mL cups and moved to chambers at the treatment temperature in either summer-like long-day (14L:10D) or fall-like short-day (8L:16D) photoperiodic conditions, which do or do not induce pupal diapause, respectively.

Temperatures within a treatment were kept constant with temperature fluctuations of ±0.5 °C or less. Pupae were sexed using the location of the gonopore and anus and the size of the last abdominal segment. Pupae were then scored for diapause by the presence or absence of pupal eyespots (Phillips and Newsom 1966). The disappearance of the pupal eyespots is concurrent with the initiation of adult development in *H. zea*, so individuals that retain pupal eyespots have suspended their development and are in diapause. Because lower temperatures slow development rates, non-diapausing pupae in lower temperatures took longer to lose their pupal eyespots and individuals were scored for diapause at an interval that was physiologically relevant to the temperature they were experiencing (between 8 and 12 days after pupation).

In preliminary experiments, we parameterized the relationship between temperature and diapause for *H. zea* by rearing larvae in different temperatures and photoperiods: 18, 19, 20, 22, and 25 °C in long-day (14L:10D) and short-day (8L:16D) conditions expected to induce direct development and diapause, respectively. Larvae were provided unlimited budworm diet, and were weighed 2 days after pupation. All larvae were scored for diapause 8–10 days after pupation. In this system, there is an interaction between larval photoperiod and temperature on pupal diapause incidence. High temperatures will override photoperiodic programming and individuals will not diapause if raised in short-day (8L:16D) conditions at or above 22 °C. Therefore, further experiments were done below 22 °C, to obtain a seasonally appropriate diapause response. Our preliminary data (Fig. S1), as well as previous work in Drosophilid flies also indicated that there was likely to be a curvilinear relationship between temperature and size (Karan et al. 1998, 1999).

### Temperature effects on size, feeding response, development time, and growth rates

To determine effects of diapause on the temperature–size relationship, larvae were placed at five different treatment temperatures at the beginning of the third instar (16–20 °C) and two photoperiods (14L:10D long day and 8L:16D short day) with 90 larvae per treatment. Larvae were fed budworm diet ad libitum and after reaching the last instar they were checked daily for pupation. Individuals in the long-day 18, 19, and 20 °C conditions were moved to a −20 °C freezer 2 days after pupation because initial trial rearings under long-day conditions showed zero diapause incidence. All other treatments were scored for diapause at the equivalent developmental stage after pupation for a given temperature, 12 days at 16 °C, 10 days at 17 and 18 °C, 9 days at 19 °C, and 8 days at 20 °C. Pupae were then frozen at −20 °C. Subsamples of all treatment groups, 352 total, were sliced in half while frozen, freeze-dried, and weighed for total mass. Individuals were then placed in perforated gelatin capsules in a Soxhlet extractor and neutral lipids were extracted with diethyl ether for 48 h (Newman et al. 1972; Tschinkel 1993). After extraction, pupae were freeze-dried again and weighed for lean mass. Nutrient reserve (fat mass) was calculated by subtracting the lean mass from the total dry mass.

We measured food consumption and waste production for 465 larvae across all treatments. Wet mass for all food provided to the larvae was measured and estimates of the dry mass of diet provided were obtained by using a standard curve of fresh diet to dry diet mass. Uneaten food and waste were collected and placed separately in a drying oven for 5 days at 40 °C. Food consumption was calculated by subtracting the dry mass of uneaten diet from the estimated dry mass of diet provided to each larva.

Development time was calculated for 351 individuals as the time in days from the beginning of the third instar, when individuals were moved to the temperature treatments, to pupation. The relative growth rate (RGR) is the average mass gain per milligram of initial mass per day. RGR was calculated for 350 individuals as [ln (pupal mass)–ln (mass third instar)]/development time (Kutcherov et al. 2011), where the mass of larvae at the beginning of the third instar was 12.3 mg, an average determined by trial rearings.

### Statistical analyses

**Fig. 1:**
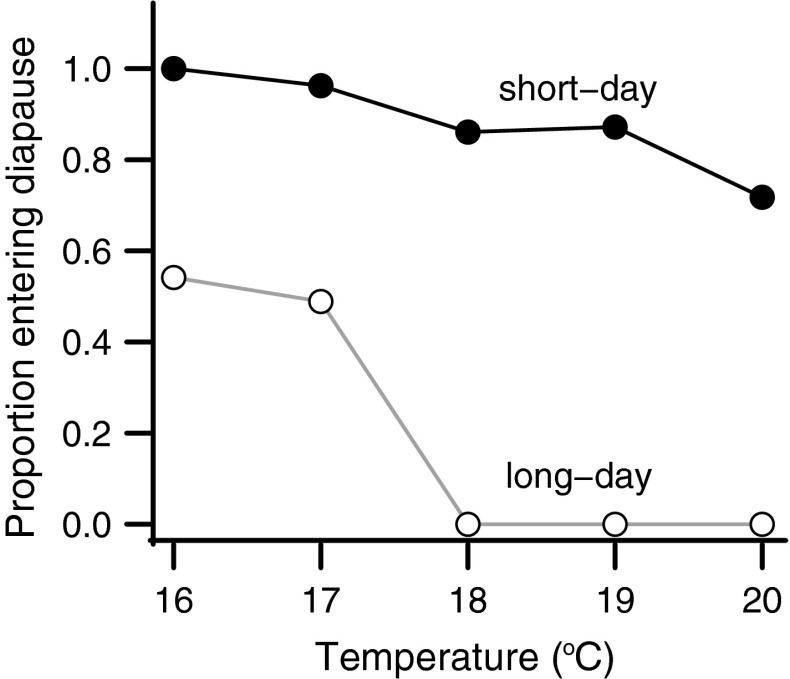
Diapause incidence in response to larval temperature and photoperiod. *Closed circles* indicate the proportion of *Helicoverpa zea* pupae entering diapause in short-day “diapause” conditions. *Open circles* indicate the proportion of pupae entering diapause in long-day “non-diapause” conditions

Statistical analyses were performed in R version 3.0.1 (R Core Team 2013). Although most individuals reared in short-day conditions entered diapause and most individuals raised in long-day conditions did not diapause, a few pupae did not perform as predicted. Too few pupae responded abnormally for statistical analyses and were therefore excluded (Fig. 1). We only included individuals that responded to long days by not diapausing and responded to short days by diapausing in our analyses.

Diapause and non-diapause classes were first analyzed separately using regression with temperature as the predictor. For each comparison, we used Akaike information criterion (AIC) model selection with the AICcmodavg package to determine if a quadratic or linear model best fit our data (Table 1). If ∆AICc < 2, i.e., the models were more similar than our threshold, we chose the model with fewer parameters as the best fit. If for a comparison diapause and non-diapause classes had the same best model (both linear or both quadratic), we then analyzed the data in a combined model with temperature, diapause status, and sex as predictors. We removed interaction effects if they were non-significant. We performed these analyses for lean mass, fat mass, food consumption, waste production, log-transformed development time, and RGR. The analysis of waste production also included food consumption as a predictor. Temperature data (and for the waste analysis, food consumption) were centered prior to analysis. In every case, with the exception of lean mass and waste production, sex was not significant and did not improve AICc, and was therefore dropped from the model. Details for combined and separate models are included in the Supplementary Information (Tables S1–S6).

**Table 1:** Model comparisons using corrected Akaike information criterion (*AICc*)

Class^a^	Type (model)^b^	Significant terms^c^	AICc
Lean mass			
Diapause	Linear (~temp)	Temp	1,427.82
	Quadratic (~temp + temp^2^)	Temp	1,428.99
Non-diapause	Linear (~temp)	Temp	1,082.83
	Quadratic (~temp + temp^2^)	Temp	1,083.60
*Combined*	*Linear (~temp* *+* *class* *+* *sex)*	*Temp, class, sex*	2,502.93
	Linear (~temp + temp^2^ + class + sex)	temp, temp^2†^, class, sex	2,501.96
Fat mass			
Diapause	*Linear (~temp)*	*Temp*	*1,397.90*
	Quadratic (~temp + temp^2^)	Temp	1,399.95
Non-diapause	Linear (~temp)	Temp	1,039.38
	*Quadratic (~temp* *+* *temp* ^*2*^ *)*	*Temp, temp* ^*2*^	*1,036.76*
Food consumption			
Diapause	Linear (~temp)	Temp	3,395.52
	Quadratic (~temp + temp^2^)	Temp, temp^2^	3,393.34
Non-diapause	Linear (~temp)	Temp	1,965.29
	Quadratic (~temp + temp^2^)	Temp, temp^2^	1,961.65
*Combined*	*Quadratic (~class* × *temp* *+* *temp* ^*2*^ *+* *class:temp* ^*2*^ *)*	*Temp, temp* ^*2*^ *, class:temp, class:temp* ^*2*^	*5,354.91*
	Linear (~class × temp)	class, temp, class:temp	5,360.39
Waste production			
Diapause	*Linear (~temp* *+* *food)*	*Food*	*2,956.94*
	Quadratic (~temp + temp^2^ + food)	Food, temp^2†^	2,955.35
Non-diapause (male)	*Linear (~temp* *+* *food)*	*Food*	*800.85*
	Quadratic (~temp + temp^2^ + food)	Food	802.08
Non-diapause, (female)	Linear (~temp + food)	Food	965.99
	*Quadratic (~food* *+* *temp* *+* *temp* ^*2*^ *+* *food:temp* ^*2*^ *)*	*Food, food:temp* ^*2*^	*961.40*
Development time			
Diapause	Linear (~temp)	Temp	−275.48
	*Quadratic (~temp* *+* *temp* ^*2*^ *)*	*Temp* *+* *temp* ^*2*^	*−300.46*
Non-diapause	*Linear (~temp)*	*Temp*	*−202.61*
	Quadratic (~temp + temp^2^)	Temp, temp^2†^	−203.46
Relative growth rate			
Diapause	Linear (~temp)	Temp	−875.57
	Quadratic (~temp + temp^2^)	Temp, temp^2^	−878.03
Non-diapause	Linear (~temp)	Temp	−583.13
	Quadratic (~temp + temp^2^)	Temp, temp^2^	−594.25
*Combined*	*Quadratic (~temp* × *class* *+* *temp* ^*2*^ *+* *class:temp* ^*2*^ *)*	*Temp, temp* ^*2*^ *, class:temp, class: temp* ^*2*^	−1,463.03
	Linear	Temp, class, class:temp	−1,446.38

Shown are the models compared for each analysis, with the best-fit model in *italic*

*Temp* Temperature

^a, b^ *(model*) indicates the actual regression model used in R, where *class* is diapause status

^c, †^ *p* < 0.10; all others listed indicate *p* < 0.05

## Results

### Lean and fat mass

**Fig. 2:**
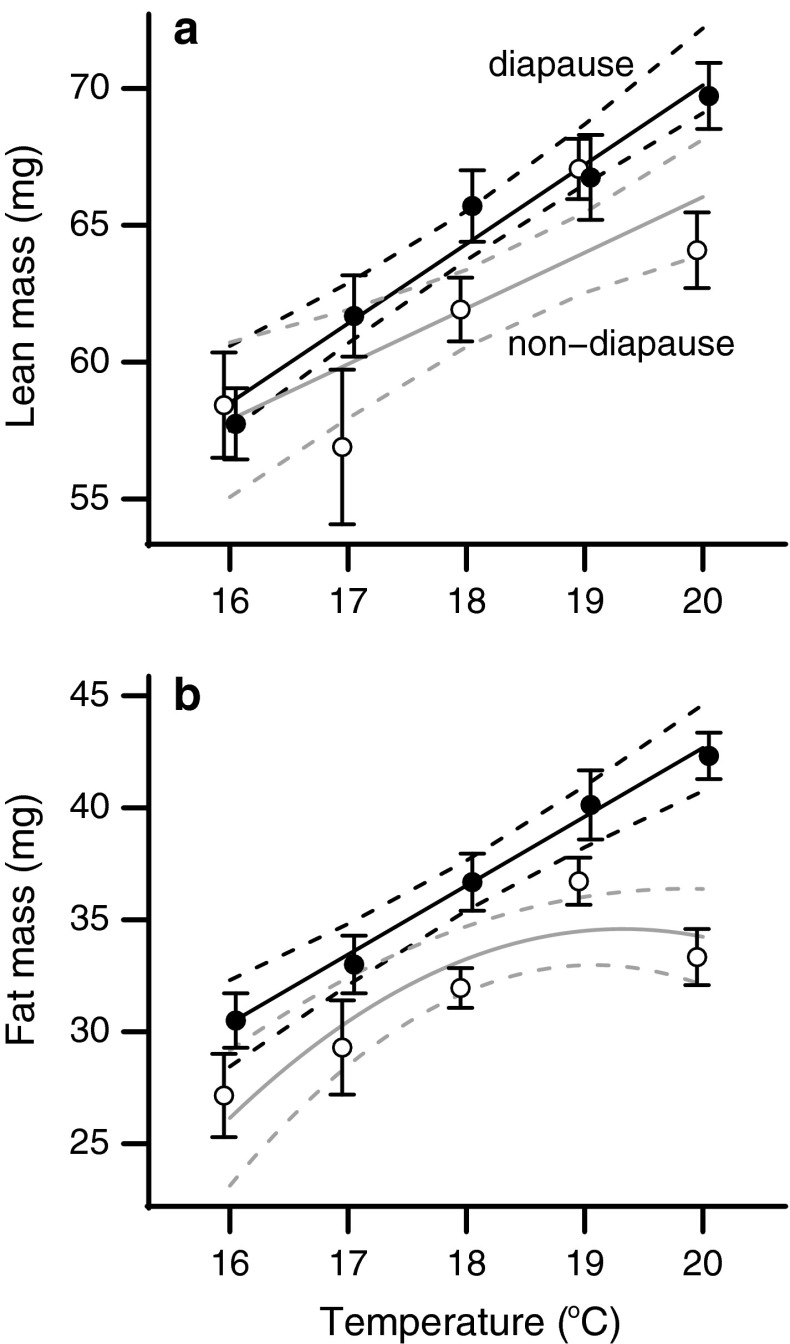
Temperature–size responses for **a** lean mass and **b** fat mass. Diapause individuals (*closed circles*) had overall greater lean mass and a linear increase in fat mass, while non-diapause individuals (*open circles*) had a quadratic relationship that leveled off at higher temperatures.* Points* show the mean response for each temperature and* error bars* indicate SEM.* Solid lines* connect the predicted value of the individual best-fit model for diapause and non-diapause groups, and* dashed lines* show the 95 % confidence interval around the predicted value

There was a clear positive, linear relationship between temperature and lean mass in pupae from both diapause and non-diapause groups (Fig. 2a). When evaluated together, diapausing pupae had greater lean mass than non-diapause pupae (*t* = 2.959, *P* = 0.003). Females were also slightly larger than males overall (*t* = −2.716, *P* = 0.007). Evaluated together or separately, in no case was a quadtratic term significant, nor did temperature interact with diapause status (Table 1; S1). For fat mass, there was a quadratic relationship between temperature and fat mass in pupae from the non-diapause group (*t* = −2.169, *P* = 0.032), whereas there was a positive, linear relationship between temperature and fat mass in pupae from the diapause group (*t* = 7.662, *P* < 0.001). As temperature increased, non-diapause individuals leveled off in their fat accumulation but diapause individuals kept increasing their fat mass (Fig. 2b). Thus, seasonal diapause programming altered lean and fat mass in fundamentally different ways across temperatures.

### Food consumption and waste production

**Fig. 3:**
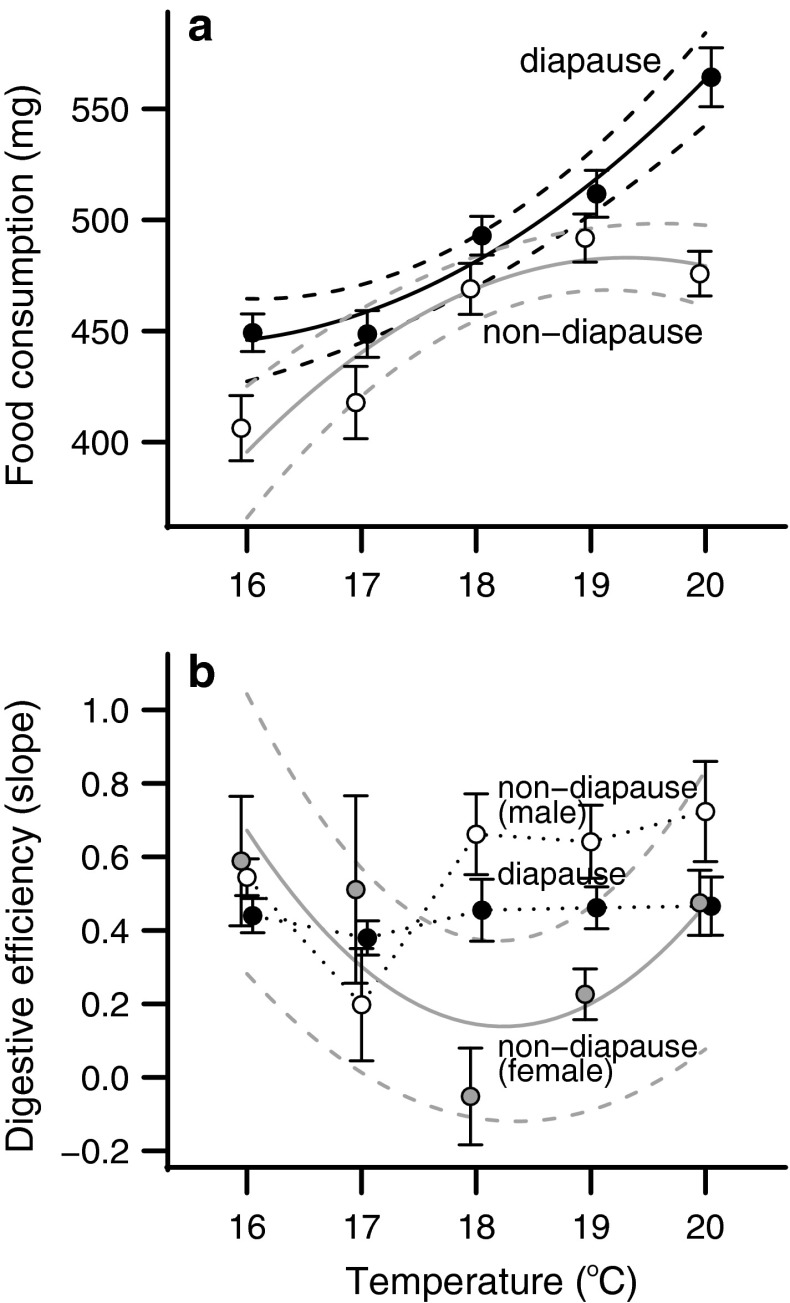
Temperature responses for **a** food consumption and **b** digestive efficiency, measured as the slope of the relationship between consumption and waste production, with lower slopes indicating greater digestive efficiency. *Points* show the mean response for each temperature and* error bars* indicate SEM. **a** Diapause individuals (*closed circles*) ate progressively more than non-diapause individuals (*open circles*), but did not differ in amount of waste produced. *Solid lines* connect the predicted value of the individual best-fit model for diapause and non-diapause groups, and* dashed lines* show the 95 % confidence interval around the predicted value. **b** Digestive efficiency for diapause individuals (*closed circles*) and male non-diapause individuals (*open circles*) was dependent on food consumption and not temperature. For female non-diapause individuals (*gray circles*), digestive efficiency depended on temperature and individuals were most efficient at intermediate temperatures. *Points* show the mean response for each temperature and *error bars* indicate SEM. *Solid lines* connect the predicted value of the individual best-fit model for diapause and non-diapause groups, and *dashed lines* show the 95 % confidence interval around the predicted value. **b** *Dotted lines* indicate groups that do not have significant temperature effects

The relationships between temperature and food consumption were quadratic for both diapause and non-diapause groups (Fig. 3a). However, the shapes of the quadratic relationship fundamentally differed between the two groups, as suggested by a clear interaction between diapause status and temperature (*t* = 3.079, *P* = 0.002). As one might expect, waste production was linearly related to food consumption. For diapausing individuals and non-diapausing male individuals, food consumption was the only factor that influenced the amount of waste produced (Table S4). However, for females that did not enter diapause, temperature altered the relationship between consumption and waste (*t* = 3.004, *P* = 0.003). Digestive efficiency can be estimated as the slope of the relationship between food consumption and waste production. In our analyses, the significant interaction between consumption and temperature in the female non-diapause group indicates that digestive efficiency changed for this group, but not for diapause or male non-diapause individuals. Female non-diapause individuals showed a curvilinear relationship that was most efficient at intermediate temperatures (Fig. 3b).

### Development time and RGR

**Fig. 4:**
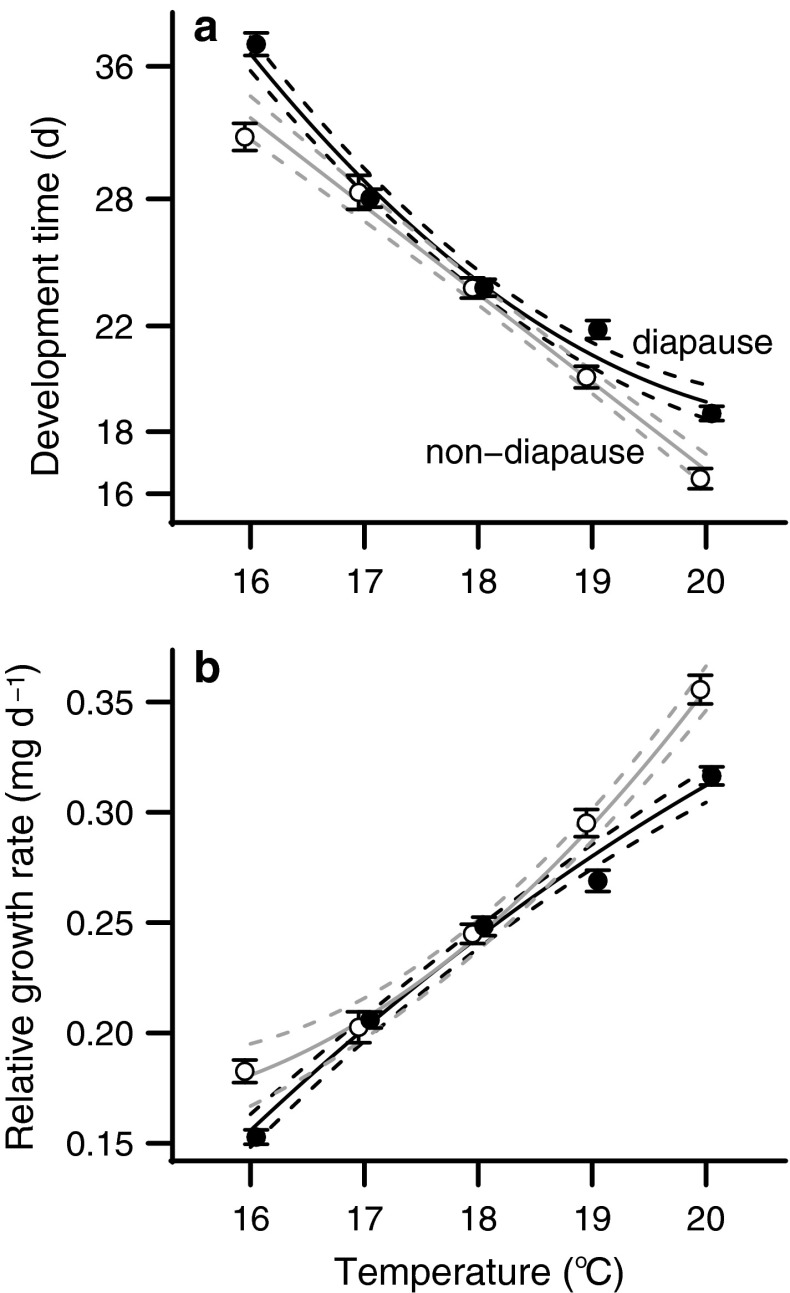
Temperature responses for **a** development time and **b** relative growth rate. As temperature increased, diapause individuals (*closed circles*) had longer development times and slower development rates than non-diapause individuals (*open circles*). **a** Development time axis in days (*d*) is on a log scale. *Points* show the mean response for each temperature and* error bars* indicate SEM. *Solid lines* connect the predicted value of the individual best-fit model for diapause and non-diapause groups, and* dashed lines* show the 95 % confidence interval around the predicted value

There was a quadratic relationship between temperature and development time in diapause-destined individuals (*t* = 5.344, *P* << 0.001), whereas there was a negative, linear relationship between temperature and development time in non-diapause individuals (*t* = −21.86, *P* < 0.001). Both groups developed faster at higher temperatures, but the diapause group has a less dramatic reduction in development time than the non-diapause group (Fig. 4a).

The relationships between temperature and growth rates were quadratic for both diapause-destined and non-diapause larvae (Fig. 4b). In a combined model there was an interaction between temperature and diapause status (*t* = −4.391, *P* < 0.001) on growth rates. Diapause-destined larvae had a lower growth rate overall and growth rate did not respond as strongly to higher temperatures in diapause-destined individuals as in non-diapause individuals.

## Discussion

Diapause programming altered the allocation of resources to pupal lean and fat mass in fundamentally different ways. For lean mass, diapausing pupae were larger overall than non-diapause pupae and lean mass increased linearly with temperature. However, the fundamental relationship between temperature and size was not different between the two groups (Fig. 2a). In contrast, the accumulation of fat mass was qualitatively different between diapause and non-diapause individuals. Diapausing individuals had greater pupal fat mass than non-diapause pupae across the entire temperature range used, and fat mass increased linearly across the rearing temperature range used (16–20 °C) (Fig. 2b). Non-diapause programmed pupae had less fat than diapausing pupae across all temperatures, and fat accumulation leveled-off at higher temperatures.

A superficial explanation of the differences in patterns of fat accumulation between diapause and non-diapause individuals is that diapausing pupae do indeed have a curvilinear relationship between fat and temperature, but that this relationship is shifted to the right and so is not observed in our experiment. However, such an interpretation does not take into account that individuals will not diapause at higher temperatures than those used in our experiment, so this hypothetical curve will not be observable in *H. zea*. Thus, we argue that the fundamental temperature–size relationship of *H. zea* pupae is altered by the diapause preparatory program expressed in growing larvae.

The observed responses in both fat and lean mass are likely caused by changes in both nutrient acquisition and developmental timing. At higher temperatures, larvae destined to diapause as pupae increased food consumption without altering their digestive efficiency (Fig. 3). Female larvae that did not enter pupal diapause did experience changes in digestive efficiency as the temperature increased but males did not. Furthermore, an increase in temperature decreased development time and increased growth rate, but this effect was not as strong on diapause-destined individuals as on non-diapause individuals (Fig. 4). The fact that diapause-destined larvae combined higher food consumption and longer development times at higher temperatures likely contributes to the shift in fat mass accumulation between diapause and non-diapause developmental programs in *H. zea*.

Studies considering the adaptive nature of temperature–size relationships often do so in the context of seasonality and explicitly include photoperiodically programmed dormancy responses (Gotthard 2008; Fischer and Karl 2010; Gotthard and Berger 2010; Esperk et al. 2013; Kivela et al. 2013). However, the relationship between body size and dormancy programming is variable. Dormancy-destined individuals sometimes having larger sizes, smaller sizes, or sizes equal to their non-dormant counterparts, a series of patterns often attributed to seasonal constraints in time for development or resource quality (Hahn and Denlinger 2007, 2011; Gotthard 2008; Gotthard and Berger 2010; Fischer and Karl 2010; Teder et al. 2010; Kivela et al. 2013). In fact, work by Nakamura (2002) and Kutcherov et al. (2011) explicitly shows that photoperiodic cues that program individuals for diapause can alter the temperature–size relationship. Although diapause-programmed individuals often contain greater nutrient reserves than their non-diapause-programmed counterparts to help sustain them through the long, non-feeding dormant period, nutrient reserves in diapause-programmed individuals can also be smaller or equal to those of non-diapause individuals (Hahn and Denlinger 2007, 2011). The literature on ectotherm dormancy responses is vast, but we were unable to find other studies like ours that concomitantly consider temperature, photoperiod, feeding, growth rates, and partition out the somatic and nutrient-storage portions of body size. Our work partitioning somatic growth from fat storage pools suggests that understanding nutrient storage relative to lean mass as part of the dormancy preparatory program can provide important complementary insights into how dormancy-inducing cues may alter the temperature–size relationship in the context of seasonal life histories.

Beyond photoperiod, many other factors that could affect resource allocation between somatic growth and nutrient storage also change seasonally. For example, the quality and predictability of food sources have been shown to both vary seasonally and alter resource-allocation patterns between somatic growth and nutrient storage across a diversity of insects (Perrin and Sibly 1993; McNamara and Houston 2008; Boggs 2009). Host plant quality in particular has been shown to alter the slope of the temperature–size reaction norm in herbivorous insects, with poor diets sometimes even completely reversing the relationship observed with high-quality diets (Stamp 1990; Diamond and Kingsolver 2010), and temperature can affect resource acquisition through both effects on diet quality and nutrient assimilation from diets (Scriber and Slansky 1981; Van Asch and Visser 2007; Coggan et al. 2011; Bauerfeind and Fischer 2013; Clissold et al. 2013; Morehouse et al. 2013). Investigating diet-dependent shifts in nutrient allocation between somatic growth and storage may reveal new proximate mechanistic insights into temperature–size relationships. These insights may ultimately provide evidence for whether the alteration of temperature–size relationships by host-plant quality is consistent with adaptive seasonal plasticity or is likely non-adaptive plasticity due to physiological constraints of growth and development.

Local adaptation in resource-allocation strategies may contribute to geographically distinct populations within species evolving different temperature–size relationships (Stillwell and Fox 2005; Kingsolver et al. 2007; Chown and Gaston 2010). One might expect that adaptive seasonal plasticity in resource-allocation patterns between somatic growth and nutrient storage, and therefore temperature–size relationships, could evolve between geographically separated populations due to local differences in the energetic demands of overwinter dormancy between sites or even through time as energy budgets during overwintering dormancy are altered by climate change (Pelini et al. 2009; Hahn and Denlinger 2011; Ragland et al. 2012; Williams et al. 2012).

It is clear that multiple ultimate selective factors and multiple proximate constraints on growth and development can affect body size, and it is the interaction of these forces that likely drive the temperature–size rule and reversals of the temperature–size rule across species, populations, and seasons. Thus it is not surprising that no single model has been satisfactory in explaining temperature–size patterns across ectotherms. In fact, whether temperature–size relationships are adaptive or non-adaptive may change from case to case, making this ubiquitous pattern of plasticity difficult to understand with a single unified model. However, we predict that including information about relative resource allocation between somatic growth and stored reserves will improve both our proximate mechanistic understanding of how and why seasonal cues that program individuals for dormancy alter temperature–size relationships and the ultimate consequences for insect performance in seasonal environments.

## Electronic supplementary material

Below is the link to the electronic supplementary material.

Supplementary material 1 (DOCX 105 kb)
