# Supplementary material for: Dormancy cues alter insect temperature–size relationships
Source: Oecologia. 2014 Sep 27;177(1):113–21. doi: 10.1007/s00442-014-3094-4 (PMC4284390; doi:10.1007/s00442-014-3094-4)
Supplement: Supplementary file 1 — Supplementary material 1 (DOCX 105 kb) [file 442_2014_3094_MOESM1_ESM.docx]

**Dormancy cues alter insect temperature-size relationships**

Sharon F. Clemmensen and Daniel A. Hahn

Supplementary Information


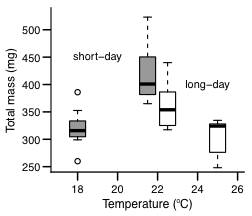


**Fig. S1**. Size response to photoperiod and temperature, preliminary data for 18, 22, and 25^o^C. Filled bars indicated short-day diapause-inducing photoperiods while open bars indicate long-day conditions that induce direct development.

**Table S1.** Lean mass regression (*) best models and combined quadratic model.

| **Lean mass combined model – linear***  **(*F*_3, 347_ = 23.35; *R*^2^ = 0.17; *P* << 0.001)** | | |
| --- | --- | --- |
| **Predictor** | ***t*-value** | ***P*** |
| (Intercept) | 79.126 | << 0.001 |
| Diapause status | 2.959 | 0.003 |
| Temperature | 7.949 | << 0.001 |
| Sex | -2.716 | 0.007 |
| **Lean mass combined model – quadratic**  **(*F*_4, 346_ = 18.37; *R*^2^ = 0.18; *P* << 0.001)** | | |
| **Predictor** | ***t*-value** | ***P*** |
| (Intercept) | 67.940 | << 0.001 |
| Diapause status | 3.063 | 0.002 |
| Temperature | 7.459 | << 0.001 |
| Temperature^2^ | -1.735 | 0.084 |
| Sex | -2.799 | 0.005 |
| **Lean mass diapause group – linear**  **(*F*_1, 197_ = 44.96; *R*^2^ = 0.19; *P* << 0.001)** | | |
| **Predictor** | ***t*-value** | ***P*** |
| (Intercept) | 104.817 | << 0.001 |
| Temperature | 6.705 | << 0.001 |
| **Lean mass non-diapause group – linear**  **(*F*_1, 150_ = ; *R*^2^ = 0.09; *P* < 0.001)** | | |
| **Predictor** | ***t*-value** | ***P*** |
| (Intercept) | 92.10 | << 0.001 |
| Temperature^2^ | 3.88 | < 0.001 |

**Table S2.** Lipid mass (*) best models and combined quadratic model.

| **Lipid mass combined model – quadratic**  **(*F*_4, 346_ = 25.45; *R*^2^ = 0.23; *P* << 0.001)** | | |
| --- | --- | --- |
| **Predictor** | ***t*-value** | ***P*** |
| (Intercept) | 42.629 | << 0.001 |
| Diapause status | 5.852 | << 0.001 |
| Temperature | 3.701 | < 0.001 |
| Temperature^2^ | -1.469 | 0.143 |
| Diapause x temperature | 1.877 | 0.061 |
| **Lipid mass diapause group – linear***  **(*F*_1, 197_ = 58.71; *R*^2^ = 0.23; *P* << 0.001)** | | |
| **Predictor** | ***t*-value** | ***P*** |
| (Intercept) | 64.174 | << 0.001 |
| Temperature | 7.662 | << 0.001 |
| **Lipid mass non-diapause group – quadratic***  **(*F*_2, 149_ = 10.5; *R*^2^ = 0.12; *P* < 0.001)** | | |
| **Predictor** | ***t*-value** | ***P*** |
| (Intercept) | 40.705 | << 0.001 |
| Temperature | 2.805 | 0.006 |
| Temperature^2^ | -2.169 | 0.032 |

**Table S3.** Food consumption regression (*) best models.

| **Food consumption combined model – quadratic***  **(*F*_5, 459_ = 22.58; *R*^2^ = 0.20; *P* << 0.001)** | | |
| --- | --- | --- |
| **Predictor** | ***t*-value** | ***P*** |
| (Intercept) | 52.686 | << 0.001 |
| Diapause status | 1.221 | 0.223 |
| Temperature | 4.207 | < 0.001 |
| Temperature^2^ | -2.250 | 0.025 |
| Diapause x temperature | 2.193 | 0.029 |
| Diapause x temperature^2^ | 3.079 | 0.002 |
| **Food consumption diapause group – quadratic**  **(*F*_2, 290_ = 38.08; *R*^2^ = 0.21; *P* << 0.001)** | | |
| **Predictor** | ***t*-value** | ***P*** |
| (Intercept) | 68.957 | << 0.001 |
| Temperature | 8.237 | << 0.001 |
| Temperature^2^ | 2.055 | 0.041 |
| **Food consumption non-diapause group – quadratic**  **(*F*_2, 169_ = 11.9; *R*^2^ = 0.12; *P* < 0.001)** | | |
| **Predictor** | ***t*-value** | ***P*** |
| (Intercept) | 60.682 | << 0.001 |
| Temperature | 2.417 | 0.017 |
| Temperature^2^ | -2.393 | 0.018 |

**Table S4.** Waste production regression (*) best models. Model with both classes omitted due to presence of significant three-way interactions.

| **Waste production diapause group – linear***  **(*F*_2, 290_ = 170.9; *R*^2^ = 0.54; *P* << 0.001)** | | |
| --- | --- | --- |
| **Predictor** | ***t*-value** | ***P*** |
| (Intercept) | 116.009 | << 0.001 |
| Food consumption | 16.240 | << 0.001 |
| Temperature | 0.721 | 0.471 |
| **Waste production non-diapause group – quadratic**  **(*F*_7, 164_ =; *R*^2^ = 0.52; *P* << 0.001)** | | |
| **Predictor** | ***t*-value** | ***P*** |
| (Intercept) | 43.880 | << 0.001 |
| Food consumption | 2.603 | 0.010 |
| Sex | 1.977 | 0.050 |
| Temperature | 1.537 | 0.126 |
| Temperature^2^ | 0.987 | 0.325 |
| Food x sex | 3.483 | 0.001 |
| Food x temperature | 2.395 | 0.018 |
| Food x temperature^2^ | 3.376 | 0.001 |
| **Waste production non-diapause group, males only – linear***  **(*F*_2, 75_ = 48.03; *R*^2^ = 0.56; *P* << 0.001)** | | |
| **Predictor** | ***t*-value** | ***P*** |
| (Intercept) | 58.334 | << 0.001 |
| Food consumption | 8.981 | << 0.001 |
| Temperature | 0.828 | 0.410 |
| **Waste production non-diapause group, females only – quadratic***  **(*F*_4, 89_ = 14.81; *R*^2^ = 0.40; *P* << 0.001)** | | |
| **Predictor** | ***t*-value** | ***P*** |
| (Intercept) | 37.135 | << 0.001 |
| Food consumption | 2.286 | 0.025 |
| Temperature | 1.138 | 0.258 |
| Temperature^2^ | 0.786 | 0.434 |
| Food x temperature^2^ | 3.004 | 0.003 |

**Table S5.** Development time quadratic regression model and (*) best models.

| **Development time combined model – quadratic**  **(*F*_5, 345_ = 299.3; *R*^2^ = 0.81; *P* << 0.001)** | | |
| --- | --- | --- |
| **Predictor** | ***t*-value** | ***P*** |
| (Intercept) | 225.425 | << 0.001 |
| Diapause status | 0.381 | 0.703 |
| Temperature | -23.076 | << 0.001 |
| Temperature^2^ | -1.789 | 0.075 |
| Diapause x temperature | 1.296 | 0.1957 |
| Diapause x temperature^2^ | 4.726 | << 0.001 |
| **Development time diapause group – quadratic***  **(*F*_2, 196_ = 446.5; *R*^2^ = 0.82; *P* << 0.001)** | | |
| **Predictor** | ***t*-value** | ***P* < *t*** |
| (Intercept) | 255.119 | << 0.001 |
| Temperature | 29.40 | << 0.001 |
| Temperature^2^ | 5.344 | << 0.001 |
| **Development time non-diapause group – linear***  **(*F*_1, 150_ = 478.0; *R*^2^ = 0.76; *P* << 0.001)** | | |
| **Predictor** | ***t*-value** | ***P* < *t*** |
| (Intercept) | 309.91 | << 0.001 |
| Temperature | -21.86 | << 0.001 |

**Table S6.** Relative growth rate regression (*) best models.

| **Relative growth rate combined model – quadratic***  **(*F*_5, 344_ = 290.1; *R*^2^ = 0.81; *P* << 0.001)** | | |
| --- | --- | --- |
| **Predictor** | ***t*-value** | ***P*** |
| (Intercept) | 71.412 | << 0.001 |
| Diapause status | -0.466 | 0.641 |
| Temperature | 24.884 | << 0.001 |
| Temperature^2^ | 4.191 | < 0.001 |
| Diapause x temperature | -3.237 | 0.001 |
| Diapause x temperature^2^ | -4.391 | < 0.001 |
| **Relative growth rate diapause group – quadratic**  **(*F*_2, 195_ = 452.2; *R*^2^ = 0.82; *P* << 0.001)** | | |
| **Predictor** | ***t*-value** | ***P* < *t*** |
| (Intercept) | 84.078 | << 0.001 |
| Temperature | 30.002 | << 0.001 |
| Temperature^2^ | -2.127 | 0.035 |
| **Relative growth rate non-diapause group – quadratic**  **(*F*_2, 149_ = 241.1; *R*^2^ = 0.76; *P <*< 0.001)** | | |
| **Predictor** | ***t*-value** | ***P* < *t*** |
| (Intercept) | 67.627 | << 0.001 |
| Temperature | 21.271 | << 0.001 |
| Temperature^2^ | 3.681 | < 0.001 |
